# The nitrobenzoxadiazole derivative MC3181 blocks melanoma invasion and metastasis

**DOI:** 10.18632/oncotarget.14690

**Published:** 2017-01-17

**Authors:** Anastasia De Luca, Debora Carpanese, Maria Cristina Rapanotti, Tara Mayte Suarez Viguria, Maria Antonietta Forgione, Dante Rotili, Chiara Fulci, Egidio Iorio, Luigi Quintieri, Sergio Chimenti, Luca Bianchi, Antonio Rosato, Anna Maria Caccuri

**Affiliations:** ^1^ Department of Experimental Medicine and Surgery, University of Tor Vergata, 00133 Rome, Italy; ^2^ Department of Surgery, Oncology and Gastroenterology, University of Padova, 35128 Padova, Italy; ^3^ Department of Laboratory Medicine, University of Tor Vergata, 00133 Rome, Italy; ^4^ Department of Drug Chemistry and Technologies, “Sapienza” University, 00185 Rome, Italy; ^5^ Department of Cell Biology and Neurosciences, Istituto Superiore di Sanità, 00161 Rome, Italy; ^6^ Department of Pharmaceutical and Pharmacological Sciences, University of Padova, 35131 Padova, Italy; ^7^ Department of Dermatology, University of Tor Vergata, 00133 Rome, Italy; ^8^ Istituto Oncologico Veneto IOV-IRCCS, 35128 Padova, Italy

**Keywords:** melanoma, 6-((7-nitrobenzo[c][1,2,5]oxadiazoles, c-Jun N-terminal kinase, antimetastatic properties, glutathione transferase P1-1

## Abstract

The novel nitrobenzoxadiazole (NBD) derivative MC3181 is endowed with remarkable therapeutic activity in mice bearing both sensitive and vemurafenib-resistant human melanoma xenografts. Here, we report that subtoxic concentrations of this compound significantly reduced invasiveness of BRAF-V600D mutated WM115 and WM266.4 melanoma cell lines derived from the primary lesion and related skin metastasis of the same patient, respectively. The strong antimetastatic activity of MC3181 was observed in both 2D monolayer cultures and 3D multicellular tumor spheroids, and confirmed *in vivo* by the significant decrease in the number of B16-F10 melanoma lung metastases in drug-treated mice. Our data also show that MC3181 affects the lactate production in the high glycolytic WM266.4 cell line. To unveil the MC3181 mechanism of action, we analyzed the ability of MC3181 to affect the degree of activation of different MAPK pathways, as well as the expression/activity levels of several proteins involved in angiogenesis, invasion, and survival (i.e. AP2, MCAM/MUC18, N-cadherin, VEGF and MMP-2). Our data disclosed both a decrease of the phospho-active form of JNK and an increased expression of the transcription factor AP2, events that occur in the very early phase of drug treatment and may be responsible of the antimetastatic effects of MC3181.

## INTRODUCTION

Malignant melanoma progresses through a multi-step process switching from dysplasia to radial growth phase, to invasive vertical growth phase, and subsequently to distant metastases. These switches implicate the breach of the basement membrane by neoplastic proliferating cells, their migration and stroma invasion to enter the vasculature, and finally their extravasation and adhesion in distant organs to form a secondary tumor [1–3]. Every phase of this complex scenario can be rate-limiting and offers potential target for therapy.

Therapeutically, one of the most recent and promising approaches is immunotherapy that involves the use of immune checkpoint inhibitors enabling the interruption of T-cell pathways responsible for immune down-regulation or tolerance, such as the anti-CTLA-4 monoclonal antibody (mAb) ipilimumab, along with the anti-PD-1 mAbs pembrolizumab and nivolumab. Another important class of drugs is represented by low molecular weight compounds interfering with signaling pathways involved in the dysregulation of cell growth and proliferation; this is the case of vemurafenib and dabrafenib, which target mutated v-raf murine sarcoma viral oncogene homolog B1 (BRAF), the selective inhibitors of extracellular signal-regulated kinase (ERK), as well as trametinib and cobimetinib targeting mitogen-activated protein kinase (MAPK)/ERK Kinase (MEK) proteins [4]. While these strategies provide a potential initial clinical benefit, they delay but not prevent patient mortality due to the ability of tumor to rapidly acquire resistance to drugs and/or to activate alternative proliferation pathways.

In contrast with current clinical trials targeting multiple signaling pathways involved in cell proliferation, we recently reported that the nitrobenzoxadiazole derivatives (NBDs) NBDHEX [6-((7-nitrobenzo[c][1,2,5]oxadiazol-4-yl)thio)hexan-1-ol] and its more water-soluble analogue, MC3181 [2-(2-(2-((7-nitrobenzo[c][1,2,5]oxadiazol-4-yl)thio)ethoxy)ethoxy)ethanol], exert a potent antitumor activity through the activation of different MAPK pathways. These compounds represent a new class of antitumor agents exhibiting an outstanding therapeutic activity together with an extremely non-toxic profile in human cutaneous melanoma mouse xenografts. NBDHEX and MC3181 inhibit glutathione transferase P1-1 (GSTP1-1), and disrupt the GSTP1-1/JNK1 and GSTP1-1/TRAF2 complexes, thereby causing prolonged tumor cell cycle arrest and apoptosis. Therefore, these drugs may constitute a new effective strategy for the treatment of BRAF-mutated human melanomas, capable of overcoming the resistance to vemurafenib [5, 6].

On this ground, we were prompted to test the antimetastatic properties of the more soluble NBD derivative, MC3181, against melanoma cells lines derived from the primary lesion (WM115) and a skin metastasis (WM266.4) of a single patient harboring a BRAF-V600D mutation.

Our data revealed that subcytotoxic doses of MC3181 exert a strong antimetastatic activity *in vitro*. To understand the mechanism(s) underling this effect, we measured the expression/activity levels of key proteins involved in melanoma cells migration and invasion, and focused on signaling pathways that modulate these proteins. Moreover, using the B16-F10 melanoma mouse model of metastasis, we demonstrated that the oral administration of MC3181 exerts a potent antimetastatic effect, significantly suppressing and/or delaying lung metastasis in the absence of detectable sign of toxicity. These data provide evidence to further support the potential of MC3181 as a novel therapeutic to treat metastatic melanoma.

## RESULTS

### Antiproliferative activity of MC3181 and NBDHEX on WM115 and WM266.4 2D monolayer cultures and 3D multicellular tumor spheroids

The antitumor efficacy of MC3181 was tested *in vitro* on 2D WM115 and WM266.4 human melanoma cell cultures, and compared with NBDHEX, temozolomide (TMZ) and vemurafenib (VMF). The concentration–response profiles (Supplementary Figure 1) fulfill the IC_50_ values reported in Table 1. Of note, the IC_50_ values calculated for MC3181 are in the low micromolar range (1.0–1.3 μM), and close to those obtained for both NBDHEX and vemurafenib (VMF), whereas TMZ is at least 600 times less effective.

**Table 1:** Evaluation of the antiproliferative (SRB assay) effects of MC3181, NBDHEX, VMF and TMZ on WM115 and WM266.4 2D monolayer cultures

Cell line	IC_50_ ± SD (μM)			
	MC3181	NBDHEX	VMF	TMZ
**WM115**	1.28 ± 0.02	1.99 ± 0.01	2.47 ± 0.19	1196 ± 103
**WM266.4**	1.07 ± 0.04	0.94 ± 0.04	0.89 ± 0.01	800 ± 5

**Figure 1:**
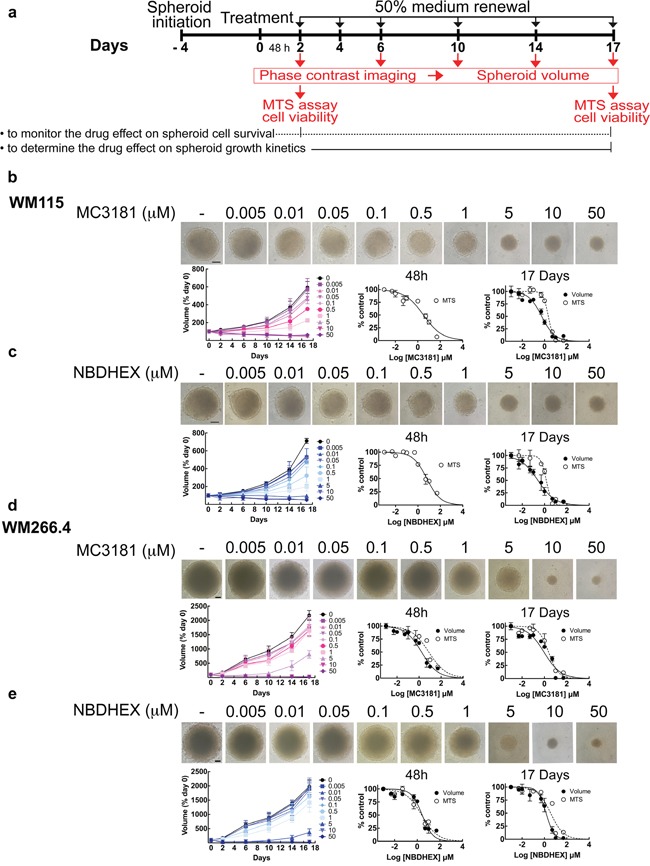
MC3181 and NBDHEX concentration-dependent inhibition of tumor spheroid growth **a**. Schematic illustration of tumor spheroid growth kinetics and compound treatment procedures. Spheroids were treated with drug or drug vehicle 4 days after cell plating (day 0); 50% medium replenishment was performed on days 2, 4, 6, 10 and 14. **b-c**. WM115 and **d-e**. WM266.4 spheroids treated with graded concentrations of MC3181 (b and d) or NBDHEX (c and e). Control spheroids were treated with vehicle. Spheroid growth kinetics (left) was evaluated by phase contrast imaging at day 2, 6, 10, 14 and 17, whereas the concentration-response curves relative to the MTS assays and spheroid volume analysis were obtained after 48 hours (center) and 17 days (right) of drug treatment. Phase contrast images (10X magnification, 3X digital magnification) correspond to 17 days treated spheroids. Scale bar: 100 μm. Values are means ± SD (n = 12).

Next, we decided to evaluate the effect of MC3181 and its parent drug, NBDHEX, on 3D multicellular tumor spheroids, which are more precise in mimicking the complex organization of tumor tissue *in vivo* [7]. Spheroids were treated with graded concentrations of MC3181 (Figure 1b and 1d) or NBDHEX (Figure 1c and 1e), and IC_50_ values were obtained by analyzing both cell viability (MTS) and growth rate. A schematic diagram for treatment schedule and analysis (cell imaging and viability assay) is shown in Figure 1a. We noticed that WM266.4 spheroids grew faster increasing their volume 25 times at the end of the experiment (day 17, Figure 1d and 1e), whilst the WM115 counterparts augmented only 8 times (Figure 1b and 1c). The IC_50_ values of MC3181 on WM266.4 spheroids were in the low micromolar range (0.5–7.7 μM, Table 2), comparable at both 48 hours and 17 days, and similar to those obtained with NBDHEX. In contrast, 48 hours treatment with both MC3181 and NBDHEX caused flaking of WM115 spheroids and formation of poorly defined contours that did not allow an accurate measurement of spheroids’ diameter (data not shown). Additionally, after 17 days of treatment, the spheroids’ viability dropped more slowly compared to the spheroids’ volume, resulting in loss of linear relationship between viability and cell number (Table 2). A similar event has been already reported and explained by the occurrence of cell cycle arrest [8].

Table 2Evaluation of the cytotoxic (MTS assay) and antiproliferative (volume analysis) effects of MC3181 and NBDHEX on WM115 and WM266.4 3D multicellular tumor spheroids

**Table:** 

IC_50_ ± SD (μM)				
	WM115			
	48 hours		17 Days	
	Volume	MTS	Volume	MTS
**MC3181**	n.d.	3.22 ± 0.19	0.53 ± 0.04	2.19 ± 0.15
**NBDHEX**	n.d.	1.44 ± 0.08	0.37 ± 0.09	6.27 ± 0.94

**Table:** 

WM266.4				
	48 hours		17 Days	
	Volume	MTS	Volume	MTS
**MC3181**	1.67 ± 0.26	7.67 ± 1.00	1.10 ± 0.23	2.41 ± 0.40
**NBDHEX**	2.84 ± 0.19	2.44 ± 0.43	1.22 ± 0.33	3.60 ± 0.51

### Effect of low concentrations of MC3181 on cell proliferation and cell cycle

Since MC3181 and NBDHEX showed comparable activity in both WM115 and WM266.4 cells, we focused on the antimetastatic efficacy of the more water soluble NBD derivative, namely MC3181. We investigated three different concentrations of MC3181 for each cell line: WM115 and WM266.4 cells were treated with MC3181 concentrations of 1.3 and 1.0 μM respectively (corresponding to their IC_50_ values), 0.26 and 0.20 μM respectively (corresponding to 1/5 of their IC_50_ values) and 0.05 and 0.04 μM respectively (corresponding to 1/25 of their IC_50_ values).

We analyzed cell proliferation and cell cycle at different time points after the addition of MC3181. A decrease in the WM115 growth rate was observed after 24 hours incubation with both 0.26 and 1.3 μM MC3181 (Supplementary Figure 2a), which corresponds to a significant arrest in the G2/M phase of the cell cycle (Supplementary Figure 2b). In contrast, WM266.4 showed only a slight decrease of cell proliferation after 48 hours treatment with the highest MC3181 dose (1.0 μM) (Supplementary Figure 2c). In accordance, the WM266.4 cell cycle was only slightly affected by treatment with 1.0 μM MC3181 (Supplementary Figure 2d).

### MC3181 treatment affects the adhesion properties of both WM115 and WM266.4 cells

The successful dissemination of tumor cells and the formation of new tumor foci require cancer cells adhesion and detachment from components of the extracellular matrix (ECM) and basement membrane (BM). Therefore, we analyzed the effect of MC3181 on the adhesion properties of WM115 and WM266.4 cells to different ECM components, i.e. type I collagen, Matrigel and gelatin [9, 10].

**Figure 2:**
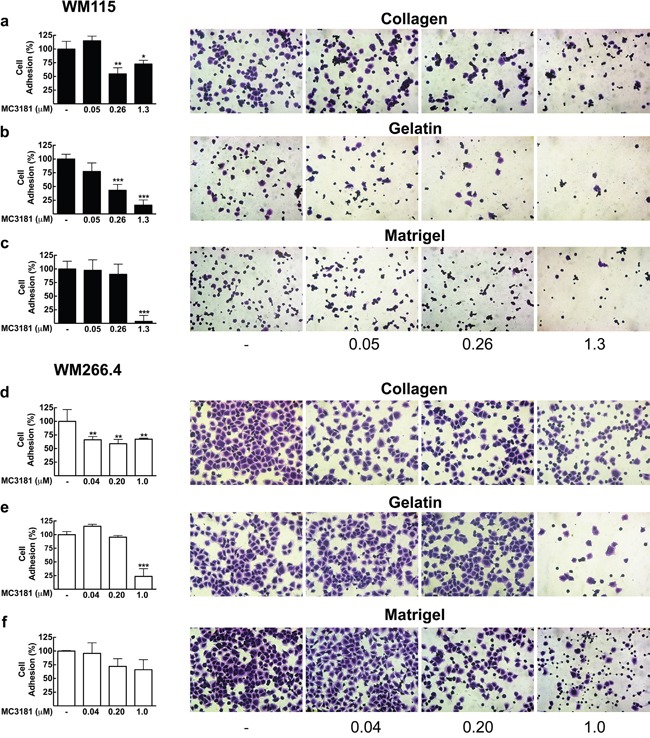
Effect of MC3181 on the adhesion of human melanoma cells to different ECM components WM115 cells and WM266.4 cells (3×10^5^/ml) were applied to individual coated wells with 200 μl of **a, d**. collagen (7.5 μg/ml), **b, e**. gelatin (0.1%) or **c, f**. Matrigel (0.2 mg/ml), in the absence and in the presence of increasing MC3181 concentrations, and incubated for 30 minutes at 37°C in 5% CO_2_. Cells were then fixed and colored with crystal violet. Images (20X magnification, 3X digital magnification) were obtained by phase contrast microscopy. The absorbance values of wells were measured at 580 nm after dissolving crystal violet with 100% methanol. The results were expressed as the mean percentage of cell adhesion ± SD versus control, repeated in triplicate; *P < 0.05, **P < 0.005 and *** P < 0.0005 *vs* control.

The primary tumor-derived WM115 cells showed poor basal adhesion on both gelatin and Matrigel (Figure 2b and 2c). The treatment with 0.26-1.3 μM MC3181 reduced to approximately 50% their adhesion to collagen (Figure 2a), whilst a concentration-dependent effect was observed on gelatin (Figure 2b), reaching an adhesion inhibition of 84% at 1.3 μM MC3181. This drug concentration was also able to abolish (96%) the adhesion of WM115 on Matrigel (Figure 2c).

The skin metastasis-derived WM266.4 cells showed excellent adhesion properties on all the substrates tested (Figure 2d-2f). However, the sensitivity to MC3181 was clearly lower than that of WM115. Indeed, 0.04 μM MC3181 was sufficient to induce 40% reduction of cell adhesion to collagen (Figure 2d), but a significant effect (80% inhibition) on gelatin adhesion was evident only with 1.0 μM MC3181 (Figure 2e). Finally, MC3181 did not show any significant inhibitory effect on tumor cell adhesion to Matrigel (Figure 2f).

### MC3181 inhibits invasion of human melanoma cells *in vitro*

**Figure 3:**
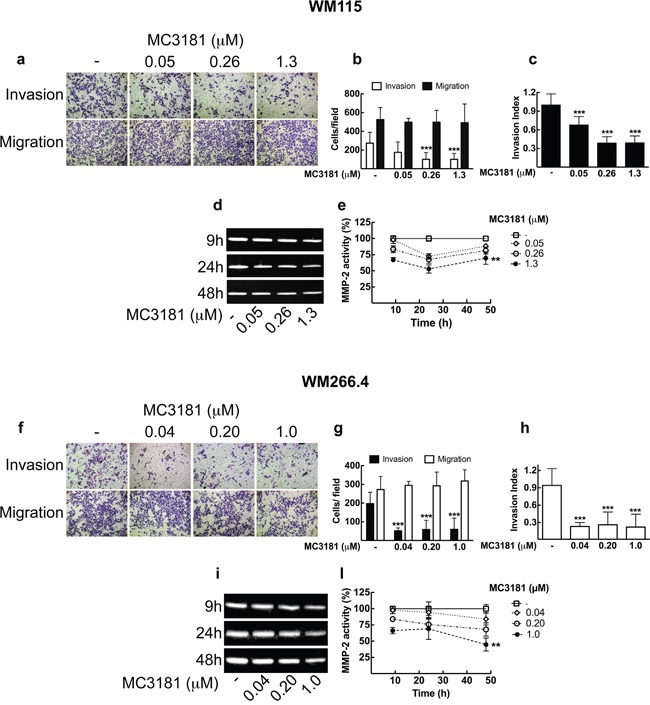
MC3181 blocks WM115 and WM266.4 melanoma cells invasion and inhibits MMP2 activity Cell lines were assayed for *in vitro* invasion and migration using Boyden chamber without coating (migration) or coated with 5 μg of Matrigel. After 48 hours of treatment with graded MC3181 concentrations, migrated and invaded cells per field were stained with crystal violet and counted. Representative phase contrast images (10X magnification, 3X digital magnification) of **a**. WM115 and **f**. WM266.4 are shown.Migrated/Invaded **b**. WM115 and **g**. WM266.4 cells. The invasion index of **c**. WM115 and **h**. WM266.4 cells was calculated as the invasion percentage of treated cells divided by the invasion percentage of non-treated cells (see equations 2 and 3 in “Materials and Methods” section). Intracellular MMP-2 activity was measured by gelatin zymography assay on **d**. WM115 and **i**. WM266.4 cells treated with graded MC3181 concentrations for up to 48 hours. ImageJ quantification of 3 independent experiments of gelatin zymography performed on **e**. WM115 and **l**. WM266.4 cells. The control has been settled as the 100%, and results were expressed as the mean percentage of MMP-2 activity ± SD *vs* control. **P < 0.005 and ***P < 0.0005 *vs* control.

Another aspect of tumor progression is the ability of tumor cells to invade basement membranes and connective tissue leading to the possible formation of distant metastases. We investigated the migratory and invasive potential of both primary tumor (WM115) and metastasis-derived (WM266.4) melanoma cells after 48 hours treatment with MC3181. This compound efficiently suppressed invasion in both cell lines, without any effect on their migration (Figure 3a, 3b, 3f and 3g). Of note, a concentration of MC3181 corresponding to 1/25 of its IC_50_ value (0.04 μM) was capable of inducing 75% reduction of WM266.4 invasion index (Figure 3h), while an equiactive concentration of MC3181 (0.05 μM) reduced the invasion index of WM115 by approximately 30% (Figure 3c). However, 60% inhibition of WM115 invasion index was obtained with 0.26 and 1.3 μM. Further, we evaluated the antimetastatic properties of MC3181 in a third tumor cell line, namely the BRAF-V600E-mutated SK-MEL-5 human melanoma cell line, which has been established from an axillary lymph node metastasis. Based on the finding that in the SRB assay MC3181 exhibited an IC_50_ value of 1.6 ± 0.1 μM towards SK-MEL-5 cells (data not shown), invasion/migration assays were carried out using the following drug concentrations: 1.60 μM (i.e., the IC_50_); 0.32 μM (i.e., 1/5 of IC_50_); and 0.06 μM (i.e., 1/25 of the IC_50_) (Supplementary Figure 3). The invasive potential of SK-MEL-5 cells was reduced by approximately 30% by 0.32 μM MC3181 whereas, a 60% inhibition was recorded at a drug concentration of 1.6 μM. Furthermore, MC3181 significantly depressed migration of SK-MEL-5 cells (Supplementary Figure 3a and 3b). Of note, a 50% reduction of cell migration was achieved with 0.32 and 1.6 μM MC3181. Since the drug inhibited both SK-MEL-5 cell invasion and migration, no change of the invasion index occurred (Supplementary Figure 3c).

### MC3181 treatment reduces MMP-2 intracellular activity

We next examined MMP-2 activity in WM115 and WM266.4 melanoma cells by gelatin zymography (Figure 3d, 3e, 3i and 3l). Densitometric analysis showed that treatment of both cell lines with equiactive concentrations of MC3181 induced a reduction of the MMP-2 activity in a concentration-dependent manner. In particular, a significant inhibition of MMP-2 proteolytic activity was observed in WM115 and WM266.4 cells treated with a concentration of MC3181 corresponding to the IC_50_ value in these cell lines (i.e., 1.3 and 1.0 μM, respectively) (Figure 3e and 3l).

### MC3181 affects invadopodia formation in WM115 and WM266.4 melanoma cell lines

**Figure 4:**
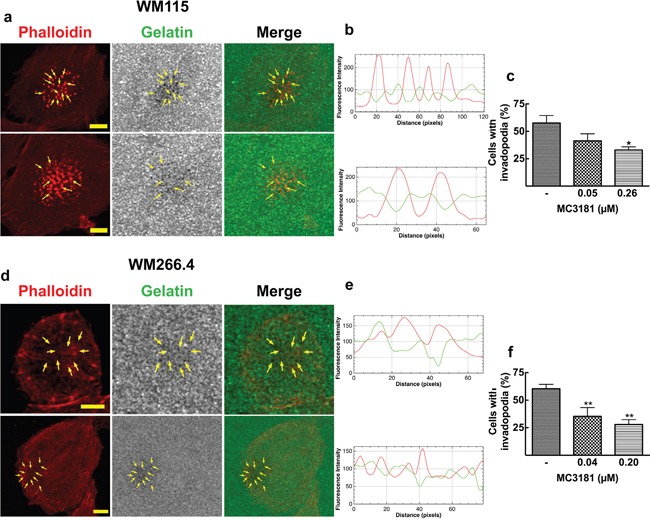
Invadopodia matrix degradation activity WM115 and WM266.4 cells were seeded on Fluorescein-Gelatin coated chamber slides for 5 hours, and then fixed and stained for TRITC-Phalloidin. **a**. WM115 and **d**. WM266.4 representative confocal images showing invadopodia as focal cytoplasmic concentrations of Phalloidin that overlap with areas of gelatin clearing (dark holes in the matrix) within the merged image, as indicated by arrows. Fluorescein-Gelatin images were pseudocolored in gray. Images were acquired with a 40X magnification oil immersion objective and a digital magnification 3X is shown. Scale bar = 10 μm. **b**. WM115 and **e**. WM266.4 representative fluorescence intensity plot showing co-localization of TRITC-Phalloidin (red line) and gelatin degradation (green line). Fluorescence intensity was measured at each image pixel along an arbitrary line that crossed through an invadopodia structure. Percentage of **c**. WM115 and **f**. WM266.4 cells with active invadopodia was obtained by counting approximately 100 cells in 3 fields. Data shown are means ± SD of three independent experiments. *P < 0.05 and **P < 0.005 *vs* control.

The strong effect of MC3181 prompted us to investigate the possible effect of the drug on the formation of invadopodia, protrusions emanating from the surface of cells and characterized by a high proteolitic activity towards the ECM. We examined the ability of WM115 and WM266.4 cells to degrade fluorescein-gelatin by observing the loss of fluorescence of the labeled gelatin and its correspondence with phalloidin structure, which identifies invadopodia puncta (Figure 4a and 4d). In addition, the presence of invadopodia was confirmed by measuring the fluorescence intensity of TRITC-phalloidin (red line) and fluorescein-gelatin (green line) along an arbitrary line that crossed through neighboring invadopodia structures. Figures 4b for WM115 and 4e for WM266.4 show a drastic decrease of the green fluorescence intensity in correspondence with an increase in the red fluorescence intensity of TRITC-phalloidin. Confocal microscopy analysis revealed a significant decrease in the number of cells with active invadopodia following treatment with concentrations of MC3181 corresponding to 1/5 and 1/25 of its IC_50_ value in these cell lines (Figure 4c and 4f). Moreover, the highest MC3181 concentrations (i.e., 1.3 and 1.0 μM for WM115 and WM266.4, respectively) completely inhibited adhesion of the cells to the fluorescein-gelatin layer. Under these conditions, it was not possible to estimate the percentage of cells with active invadopodia.

### Effects of MC3181 treatment on intracellular metabolome of WM115 and WM266.4 melanoma cells

A comprehensive analysis of the effects of intracellular metabolome was carried out by quantitative ^1^H-NMR spectroscopy on WM115 and WM266.4 cells following MC3181 treatment for 48 hours.

As shown in Supplementary Figure 4, we found a progressive decrease in the intracellular levels of lactate in the high glycolytic WM266.4 cells, while we did not found other metabolic changes in the same cells (see Supplementary Tables 1A and 1B). No evident effects on metabolome were found in WM115 cells treated at both low and high concentrations of MC3181.

### Analysis of the effect of MC3181 treatment on the mRNA expression of a panel of genes involved in melanoma progression and metastatization

Qualitative RT-PCR was carried out to characterize WM115 and WM266.4 cell lines for the expression of a panel of genes, including the pro-angiogenic factors vascular endothelial growth factor (VEGF) and the basic fibroblast growth factor (FGF2); the matrix-metallo-proteinases 2 and 9 (MMP-2 and MMP-9); the cell-cell adhesion molecules E-cadherin (CDH1), N-cadherin (CDH2), Ve-cadherin (CDH5), and both the long and short isoforms of the endothelial antigen MCAM/MUC18 [11]. To validate our expression panel, we used two melanoma cell lines as positive control (M10 and M14) and β_2_-microglobulin as housekeeping.

**Figure 5:**
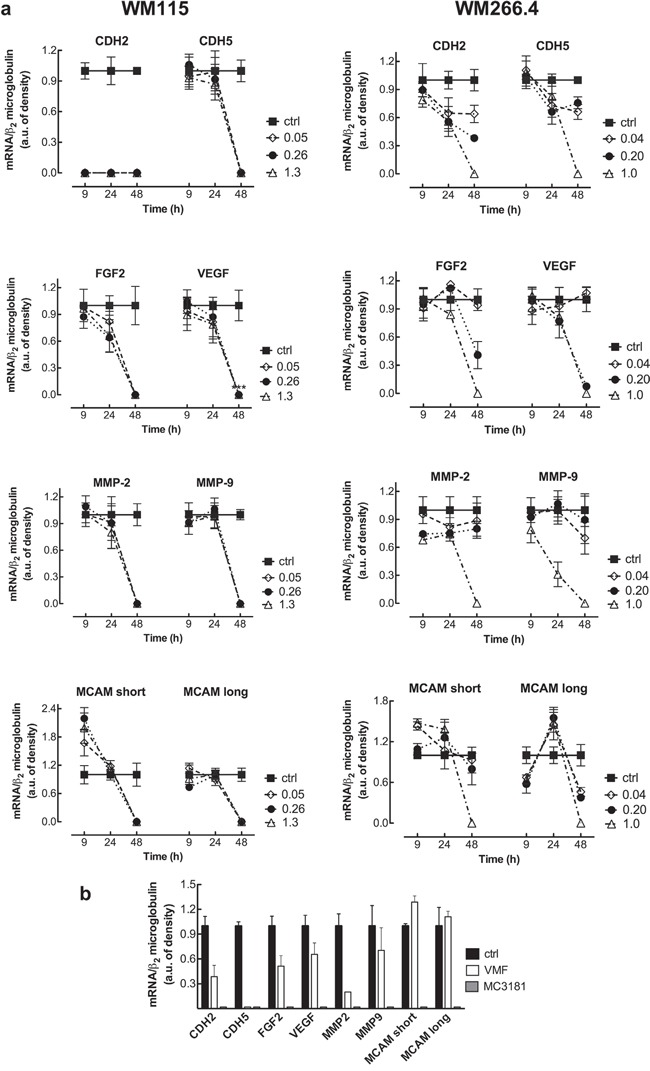
RT-PCR analysis of the mRNA expression of cancer progression related genes **a**. Total RNA from untreated and MC3181-treated cells was extracted, reverse-transcribed, amplified by PCR, and submitted to electrophoresis on a 1.8% agarose gel (see “Materials and Methods” section). The graphs show the relative intensity of the PCR products vs β2-microglobulin, obtained by the ImageJ analysis software: -■-ctrl; -◇- 0.05 and 0.04 μM MC3181 (WM115 and WM266.4, respectively); -●- 0.26 and 0.20 μM MC3181 (WM115 and WM266.4, respectively) and -△- 1.3 and 1.0 μM MC3181 (WM115 and WM266.4, respectively). **b**. Relative intensity of the PCR products from WM266.4 cells treated for 48 hours with equiactive concentrations of MC3181 and VMF (1.0 μM *vs* 0.9 μM, respectively).

RT-PCR documented mRNA expression in all the melanoma cell lines analyzed (WM115, WM266.4, and the positive controls M10 and M14) for VEGF, FGF2, CDH2, CDH5, MMP-2, MMP-9, and the two isoforms of MCAM/MUC18. The mRNA expression levels of all the genes tested in WM115 and WM266.4 cell lines are shown in Figure 5a, and a representative gel is reported in Supplementary Figure 5. Afterwards, we analyzed the mRNA expression of these genes following treatment with equiactive concentrations of MC3181 (corresponding to its IC_50_ value, 1/5 and 1/25 of its IC_50_ value in these cell lines). CDH5, VEGF, FGF2, MMP-2, MMP-9 and both the MCAM/MUC 18 Long and Short isoforms mRNA were still present at 24 hours in WM115 cells, while they drastically decreased at 48 hours after treatment with all the MC3181 concentrations tested (Figure 5a, left). Interestingly, 9 hours of drug exposure were sufficient to induce a drastic decrease of the CDH2 mRNA (Figure 5a, left). Conversely, a strong reduction of mRNA expression of all genes could be observed in WM266.4 cells after 48 hours of treatment only with the highest concentration (1.0 μM) of MC3181 (Figure 5a, right). We did not detect the presence of CDH1 mRNA in both WM115 and WM266.4 cell lines, either before or after MC3181 treatment (data not shown).

Finally, we decided to perform a preliminary comparison of the efficacy of equiactive concentrations of MC3181 and VMF, corresponding to their IC_50_ values (1.0 and 0.9 μM, respectively), on the mRNA expression. Interestingly, we found that MC3181 treatment outperformed VMF in the suppression of the genes analyzed after 48 hours, with the only exception of CDH5 (Figure 5b).

### MC3181 inhibits WM266.4 3D-multicellular tumor spheroid invasion into collagen type I

**Figure 6:**
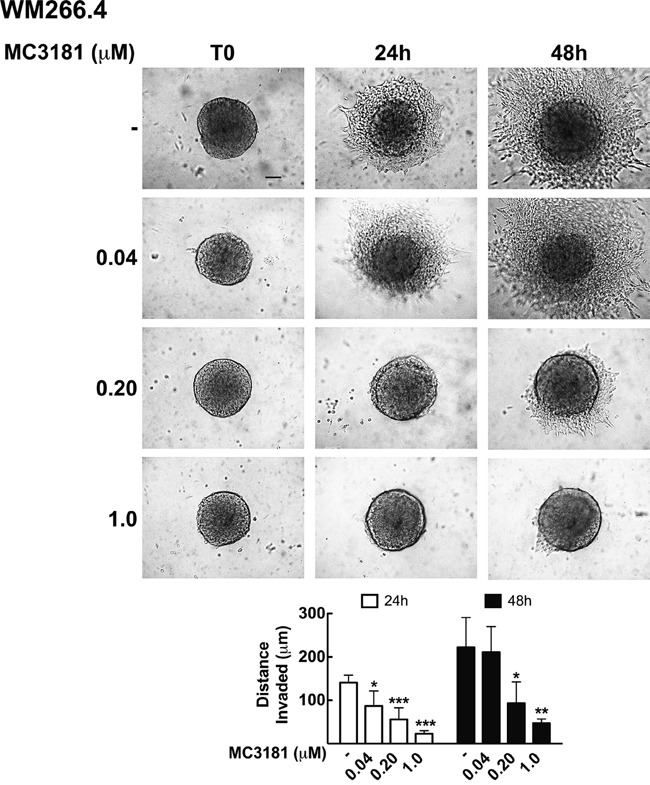
MC3181 inhibits WM266.4 spheroids invasion into type I collagen 3D melanoma spheroids were embedded in a collagen matrix and treated with 0.04, 0.20 and 1.0 μM MC3181. The distance invaded by WM266.4 cells was monitored by phase contrast imaging (10X magnification, 3X digital magnification), over a 48 hours incubation period. Scale bar: 100 μm. The graph shows the mean distance invaded by spheroids ± SD; sample number: n = 10 for every condition; *P < 0.05, **P < 0.005 and ***P < 0.0005 *vs* the respective time-matched control.

Tumor invasion was further analyzed in WM115 and WM266.4 cells grown as 3D-multicellular tumor spheroids and embedded in type I collagen. Under these experimental conditions, spheroids formed by WM115 cells were not able to invade the collagen matrix, and therefore subsequent experiments were performed only on WM266.4 cells. Also in this case, the compound was able to significantly reduce cells invasion into collagen, in a dose dependent manner (Figure 6). Twenty-four hour treatment with 0.04, 0.20 and 1.0 μM MC3181 reduced the distance invaded by WM266.4 cells by about 40, 60 and 80%, respectively, compared to the control. The effect obtained with 0.20 and 1.0 μM MC3181 remained constant over the 48 hours incubation period, while spheroids treated with 0.04 μM MC3181 showed a recovery of their invasive potential.

### MC3181 down-regulates the phospho-activation of c-Jun N-terminal kinase (JNK) and p38

**Figure 7:**
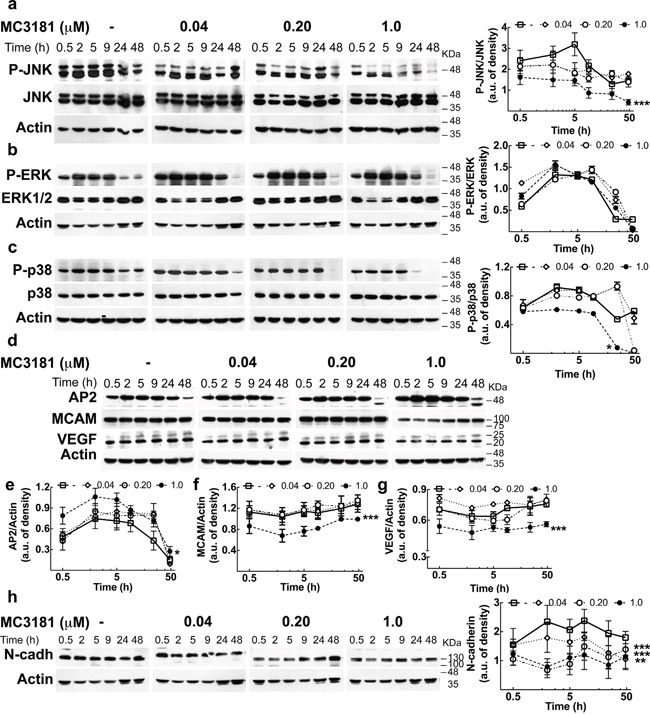
MC3181 causes a persistent decrease of JNK/p38 phospho-activation and of the expression of proteins involved in melanoma invasion Immunoblot and densitometric analysis of **a**. P-JNK, **b**. P-ERK, **c**. P-p38 in absence (-□-) and in presence of MC3181 0.04 μM (-◊-), 0.20 μM (-○-) and 1.0 μM (-●-). The data revealed a significant decrease of P-JNK after only 30 minutes treatment with 1.0 μM MC3181 (***P < 0.0005 *vs* control). A sustained decrease of the phospho-active form of p38 was also observed at the highest MC3181 concentration (1.0 μM, *P < 0.05 *vs* control). Immunoblot of **d**. AP-2, MCAM/MUC18 (CD146) and VEGF. Actin was used as loading control. Densitometric analysis revealed a prolonged (up to 24 hours) increase of **e**. AP2 expression after treatment with 1.0 μM MC3181 (*P < 0.05 *vs* control) that paralleled the persistent decrease of the expression levels of **f**. MCAM/MUC18 and **g**. VEGF (***P < 0.0005 *vs* control) caused by 1.0 μM MC3181. All subtoxic concentrations of MC3181 (from 0.04 to 1.0 μM) were also able to efficiently reduce the **h**. N-cadherin expression up to 48 hours in WM266.4 cells (**P < 0.005 and ***P < 0.0005 *vs* control). Phosphorylated and non-phosphorylated proteins levels were quantitated by densitometry and normalized to their respective β-actin bands; data, presented as arbitrary units, represent means ± SD of three independent experiments. The x-axis is in logarithmic scale.

To gain insight about the molecular mechanisms, we analyzed the effect of MC3181 treatment on proteins involved in WM266.4 cell invasion. Since activation of the MAPK/ERK pathway is a frequent event in tumorigenesis, we investigated the activation level of JNK, ERK and p38. We found that MC3181 was able to induce a significant reduction of P-JNK levels in the metastasis-derived melanoma cell line, starting within 30 minutes after addition of the drug (Figure 7a). This reduction persisted for 48 hours in the presence of 1 μM MC3181. Of note, when WM266.4 cells were treated with higher MC3181 concentrations (i.e. 2.0 and 4.0 μM) we observed a persistent JNK activation that paralleled an increase of apoptotic cells, as previously reported (Supplementary Figure 6) [5, 6]. Phospho-activation of p38 was also significantly affected by 1.0 μM MC3181 up to 48 hours (P-p38, Figure 7c). As regards ERK activation, the levels of P-ERK in drug treated cells were not significantly different from those of control cells, at all of the investigated MC3181 concentrations (Figure 7b).

### Autophagy is not involved in the anti-metastatic effect of MC3181

As autophagy seems to be involved in tumor cell motility and invasion [12], we investigated the impact of subtoxic concentrations of MC3181 on the basal autophagy of WM266.4 cells. Western blot analysis showed that a 48-hours treatment with low concentrations of MC3181 did not significantly affect the levels of the autophagosome-associated LC3-II protein (Supplementary Figure 7). Thus, the anti-metastatic activity of MC3181 is not attributable to autophagy modulation.

### MC3181 reduces the expression of proteins involved in tumor invasion and angiogenesis and increases the level of the AP2 transcription factor

Then, we checked the expression levels of several proteins involved in the extravasation process. Treatment of WM266.4 cells with 1.0 μM MC3181 caused a sustained increase of the transcription factor AP2 (activating enhancer binding protein 2), starting within 30 minutes after addition of the drug (Figure 7d and 7e). Furthermore, 1.0 μM MC3181 caused a significant and sustained decrease in the expression of the adhesion molecules MCAM/MUC18 (CD146), VEGF and N-cadherin (Figure 7d, 7f, 7g and 7h). Of note, the expression of the latter protein was significantly affected also by lower concentrations of MC3181.

### MC3181 oral administration reduces melanoma-induced lung metastases

**Figure 8:**
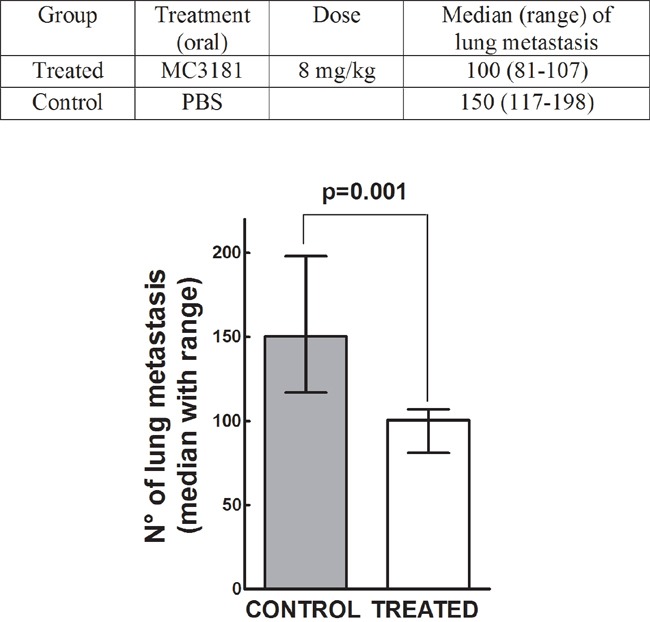
Antimetastatic effect of MC3181 *in vivo* On day 0, C57BL/6 mice were injected i.v. with 1×10^5^ Syngeneic B16-F10 melanoma cells. Eight hours later, they were randomly assigned to an experimental group (n = 6) and received orally MC3181 dissolved in PBS. Control group received PBS only. Mice were sacrificed on day 14, and lung metastatic nodules counted with the aid of a dissecting microscope. P = 0.001.

To assess the potential antimetastatic effect of MC3181 *in vivo*, B6 mice were injected i.v. with syngeneic melanoma cells at day 0, and treated 8 hours later, and subsequently on a daily base, with an oral administration of the drug. Two weeks after tumor cell injection, animals were sacrificed, lungs explanted and metastases counted. MC3181 treatment reduced by more than 30% the number of metastatic lung nodules, as compared to untreated mice (Figure 8), thus supporting the concept that the drug may be particularly active in preventing melanoma invasion and metastasis.

## DISCUSSION

The ability of cancer cells to migrate, invade, and metastasize to other organs represents the most lethal aspect of melanoma, as well as the major hurdle to successful therapeutic intervention.

In the present study, we demonstrated that subtoxic concentrations of MC3181 are effective in reducing the invasiveness of both two BRAF-V600D-mutated melanoma cell lines (namely, WM115 and WM266.4) as well as of the BRAF-V600E-mutated human melanoma cell line SK-MEL-5.

We previously reported [6] the high antiproliferative activity of MC3181 against a wide panel of melanoma cells *in vitro*. Here, we confirmed this potent and concentration-dependent ability in both 2D monolayer and 3D multicellular spheroids cultures. Moreover, since cell adhesion is a fundamental step involved in the physiological processes of proliferation, invasion, as well as the pathology of neoplastic transformation and metastasis, we were prompted to investigate the capacity of MC3181 to hinder this phenomenon in both 2D and 3D-cultures. The drug successfully affected the adhesion properties of cell cultures, and interfered also with invadopodia formation. Moreover, MC3181 caused a concentration-dependent decrease of lactate levels in the high glycolytic WM266.4 cells. MC3181 acted selectively to inhibit lactate formation without affecting intracellular metabolites involved in glucose, phospholipid and energetic metabolism of this cell line. Indeed, an increase in intracellular lactate (Warburg metabolic phenotype) causes a consistent acidification of the tumor microenvironment triggering epithelial–mesenchymal transition (EMT) in melanoma cells and promoting invasion and metastasis through expression, activation and secretion of proteolytic enzymes [13]. Consequently, the ability of MC3181 to counteract the formation of an acidic microenvironment may represent a crucial step in the suppression of metastatic phenotype of WM266.4 cells. Overall, these data are paralleled by the *in vivo* antimetastatic activity observed against the highly metastatic B16-F10 murine melanoma cells.

To gain insight into the mechanisms of action underlying these results, we investigated the effects of MC3181 on the expression of genes involved in melanoma progression and metastatization, at both mRNA and protein levels. In particular, we focused our analysis on the transcription factor AP2, known to regulate the developmental choice between growth and differentiation in several embryonic tissues, and whose expression levels inversely correlate with melanoma progression in human primary melanoma specimens [14–21]. Of note, MC3181 caused an early increase in the expression levels of AP2 in the metastatic cell line, together with a decrease of N-cadherin, MCAM/MUC18 and VEGF. In fact, this transcription factor acts as a tumor suppressor, and its loss results in the up-regulation of the expression of several genes involved in the acquisition of the metastatic phenotype [16, 22–24].

In particular, N-cadherin favors the interaction of melanoma cells with other cell types expressing N-cadherin, such as fibroblasts or vascular endothelial cells [25, 26], thus fostering the access of tumor cells to the vasculature and the formation of metastases [27]. This process is also promoted by the proteolytic activity of matrix metalloproteinase MMP-2, which degrades collagen IV [28], a major constituent of ECM, and favors melanoma cells to cross the basal lamina and migrate to their secondary sites of growth. Indeed, we showed here that MC3181 also induced a significant reduction of MMP-2 activity. These data are also supported by the evidence that MC3181 treatment decreases the number of cells with active invadopodia. Thus, MC3181 treatment appears to force melanoma towards a less aggressive phenotype lowering the expression of several proteins involved in invasion and angiogenesis.

We previously reported that cytotoxic amounts of NBDHEX and MC3181 can disrupt the interaction between GSTP1-1 and TRAF2, leading to a prolonged activation of different MAPK pathways and eventually cell death [6, 29]. This is in contrast with the fact that activation of MAPKs is involved in the control of cell migration and invasion by regulating the expression and activation of MMPs [30]; in particular, the activating mutations in the Ras/Raf/MEK/ERK proteins result in constitutive signaling that promote the oncogenic behavior of melanomas. Moreover, the MAP kinase JNK plays a role in the acquisition of the metastatic phenotype, acting at multiple levels. JNK interacts with and phosphorylates paxillin leading to an increased formation of invadopodia in gliomas [31], and JNK inhibition in melanoma by α-solanine significantly reduces cell migration and invasion [32]. Surprisingly, we found that subtoxic amounts of MC3181 not only did not activate ERK but also caused a significant and sustained decrease of the phospho-active forms of JNK and p38. This event occurred in the very early phase of drug treatment, and paralleled the increase in the AP2 expression. Nonetheless, the potential involvement of alternative MC3181 targets cannot be ruled out. However, when the amount of MC3181 was increased to concentrations higher than its IC_50_ value, we confirmed the previously reported evidence of a persistent phospho-activation of JNK driving cell death.

The molecular alterations reported above may also explain the effect of MC3181 treatment on the amount of lactate present in the metastatic WM266.4 cancer cells. Gao et al. [33] found higher nuclear levels of pyruvate kinase isoform M2 (PKM2) in WM266.4 cells, as compared to those recorded in the parent WM115 cells. Nuclear PKM2 could act as a coactivator of β-catenin to induce the expression of c-Myc, which in turn promotes the expression of glycolytic genes. In particular, c-Myc transcriptionally induces the expression of both glucose transporter 1 and lactate dehydrogenase A, which are required for glucose uptake and conversion of pyruvate to lactate, respectively [34]. c-Myc transcription is also regulated by the JNK/AP1 pathway [35], and the inhibition of this pathway causes a decrease in the lactate production [36]. Furthermore, the transcription factor AP-2 directly interacts with c-Myc and inhibits its binding to DNA [37]. Therefore, we may hypothesize that both inhibition of JNK phospho-activation and increment of AP2 levels may be responsible for the decrease of lactate production in WM266.4 cells upon treatment with MC3181.

Overall, the results of the present study are particularly relevant considering the high unmet need for more effective and safer systemic therapies for metastatic melanoma.

## MATERIALS AND METHODS

### Drugs

NBDHEX and MC3181 were synthesized as previously reported [6, 38]. For *in vitro* studies, NBDHEX, MC3181, temozolomide (TMZ; Sigma-Aldrich, Milan, Italy) and vemurafenib (VMF; Selleckem, Munich, Germany) were dissolved in DMSO. Before use, each compound was diluted to the appropriate concentration in complete culture medium; the final DMSO concentration never exceeded 0.05% (v/v). For *in vivo* studies, MC3181 was dissolved in phosphate-buffered saline (PBS).

### Cell culture and treatment

The human melanoma cell lines WM115, WM266.4, SK-MEL-5, M10, and M14; the Ehlers-Danlos syndrome (EDS) derived fibroblast cell line; and the mouse melanoma cell line B16-F10 were obtained from the American Type Culture Collection (ATCC, Manassas, VA). WM115, WM266.4, M10, and M14 cell line were cultured in RPMI-1640 (EuroClone, Milan, Italy) whilst SK-MEL-5 were cultured in DMEM/F12 (Sigma-Aldrich) supplemented with 10% fetal bovine serum (FBS, v/v) (EuroClone), 2 mM L-glutamine, 100 U/ml penicillin and 100 mg/ml streptomycin (Lonza, Basel, Switzerland). B16-F10 cells were cultured in DMEM supplemented with the above-mentioned reagents and 50 mM β-mercaptoethanol. All cell lines were maintained at 37 °C in a 5% CO_2_ humidified atmosphere.

For *in vitro* antiproliferative studies, WM115 (2.5×10^4^ cells/cm^2^), WM266.4 cells (1.25×10^4^ cells/cm^2^) and SK-MEL-5 (2.5×10^4^ cells/cm^2^) were seeded in 96-well plates and the IC_50_ values for each compound was then evaluated by the SRB assay as previously reported [5, 6].

The effects of MC3181 on cell growth and cell cycle were evaluated in WM115 and WM266.4 cell lines (2.5×10^4^ and 1.25×10^4^ cells/cm^2^, respectively) seeded in 75 cm^2^ flask (Corning B.V. Life Sciences, Amsterdam, Netherlands). Forty-eight hours after plating, cells were exposed to equiactive concentrations of MC3181 (0.05, 0.26 and 1.3 μM for WM115, and 0.04, 0.20 and 1.0 μM for WM266.4), harvested at different time points, counted using a Neubauer Chamber (after 1:1 dilution in Trypan Blue), and analyzed by a FACSCalibur instrument (BD Bioscence, San Jose, CA, USA). Flow cytometric data were analyzed by FlowJo 8.8.6 software (Tree Stare, Inc, Ashland, OR, USA).

### Cell adhesion assay

A 48-well plate was coated with 200 μL of collagen (7.5 μg/ml), gelatin (0.1%), or Matrigel (0.2 mg/ml), and incubated for 2 hours at room temperature (RT). After PBS washes, plates were blocked by incubation for 30 minutes at 37°C in cell culture medium. Human melanoma cells (5×10^4^ cell/well) were suspended in complete medium in the absence or presence of graded concentrations of MC3181, then plated onto the pre-coated wells, and allowed to adhere to the different substrates for 30 minutes at 37°C. Non-adhering cells were washed off with PBS, and the adhering cells were fixed with cold methanol (Sigma-Aldrich) and stained with 0.5% crystal violet. Digital photos of 3 random fields were taken at magnification 20X (3X digital camera) with an inverted microscope (Nikon ECLIPSE TS100). After imaging, the absorbance of the solubilized dye (in 100% methanol) was measured at 580 nm. The data are expressed as the mean percentages ± SD from at least three independent experiments.

### Gelatin zymography

WM115 and WM266.4 cells (2×10^6^) were treated with different amounts of MC3181 (0.05, 0.26 and 1.3 μM for WM115, and 0.04, 0.20 and 1.0 μM for WM266.4) for 9, 24 or 48 hours, and then lysed in Lysis Buffer [25 mM Tris-HCl (pH 7.5), 100 mM NaCl, 1% Nonidet-P-40, 1 mM PMSF, pH 7.5] (Sigma-Aldrich, Milan, Italy). After determination of protein concentration by the Lowry colorimetric assay, 20 μg of proteins were loaded, under non-reducing conditions, on 10% SDS-polyacrylamide gels containing 0.1% gelatin (Serva ELECTROPHORESIS, Heidelberg, Germany). After electrophoresis, gels were incubated in the Renaturing Solution [2.5% Triton X-100 (Sigma-Aldrich)] for 30 minutes at room temperature, and then incubated in the developing buffer [50 mM Tris-HCl (pH 7.8), 200 mM NaCl, 5 mM CaCl_2_, 0.02% Triton X-100] overnight, at 37°C. Gels were then stained with 0.5% Comassie blu R250 (Sigma-Aldrich) for 1 hour, and destained with 10% methanol and 5% acetic acid (Sigma-Aldrich). Activity was obtained by analysing the clear areas in the gels with the ImageJ software. The data are expressed as the mean percentages ± SD from three independent experiments.

### Invadopodia assay and confocal laser scanning microscopy

WM115 and WM266.4 cells (2.0×10^4^/cm^2^) were seeded on 8-well chamber slides previously coated with Fluorescein-Gelatin following manufacturer's protocol (QCM™ Gelatin Invadopodia Assay Green, Millipore). After 5 hours incubation in absence and in presence of graded MC3181 concentrations (0.05, 0.26 and 1.3 μM for WM115, 0.04, 0.20 and 1.0 μM for WM266.4), cells were fixed with 3.7% formaldehyde, washed in PBS, permeabilized by fluorescent staining buffer (2% blocking serum and 0.25% Triton X-100 in DPBS without Ca^2+^ and Mg^2+^), and then stained for TRITC-Phalloidin and DAPI following manufacturer's instruction. Fluorescence was detected using a Fluoview 1000 Olympus (Opera Zerbo, Milan, Italy) system equipped with an Olympus IX-81 inverted microscope. Acquisitions were performed with a 40X magnification oil immersion objective (NA 1.42, WD 0.15 mm). Cells with active invadopodia were defined as cells characterized by dot like structures. The Z-optical section series, obtained beginning from the nuclear apex and progressing down in 0.48 μM (at least 22 planes), were converted to maximum projection images to avoid subjectivity in the choice of the plane to be analysed. Gelatin degradation was visualized as darker areas on the coverslip due to proteolytic removal of the Fluorescein-Gelatin. Almost 100-150 cells/sample were analysed in merged images by the ImageJ software.

### High-resolution ^1^H-NMR analyses of cell extracts

WM115 (2.5×10^4^ cells/cm^2^) and WM266.4 cells (1.25×10^4^ cells/cm^2^) were plated in 150 cm^2^ flasks and, 48 hours after seeding, were treated with equiactive MC3181 concentrations (0.26 and 1.3 μM for WM115, 0.20 and 1.0 μM for WM266.4). Cells were trypsinized 48 hours after treatment, counted, and assessed for viability and membrane integrity by trypan blue staining. Following washes with ice-cold PBS, cell pellets were resuspended in 0.5 ml of ice-cold twice-distilled water. Aqueous extracts (from 10 × 10^6^ cells/sample) were prepared in ethanol 70% according to an established protocol [39]. Briefly, samples were ultra-sonicated at 20 kHz by a MSE ultrasonic disintegrator Mk2 (Crawley) and centrifuged at 14000 x g for 30 min. Supernatants were lyophilized twice in a RVT 4104 Savant lyophilizer (Mildford), and the residue resuspended in 0.7 ml D2O (Sigma-Aldrich, St. Louis, MO, USA) containing 0.1 mM 3-(trimethylsilyl)-propionic-2,2,3,3-d4 acid sodium salt (TSP) as internal standard. High-resolution NMR experiments (25°C) were performed at 9.4T (Bruker AVANCE). ^1^H-NMR spectra of cell extracts were acquired using 90° flip angle, 30 s repetition time, 32K time domain data points and 128 transients [39, 40].

### RNA isolation and reverse transcriptase-polymerase chain reaction (RT-PCR)

WM115 and WM266.4 cells were plated in 75cm^2^ flask (2.5×10^4^ cells/cm^2^ and 1.25×10^4^ cells/cm^2^, respectively). Forty-eight hours after seeding, cells were incubated with increasing concentrations of MC3181 for 9, 24, and 48 hours. Two human melanoma cell lines (M10 and M14) were used as positive control, while the negative counterpart is represented by the Ehlers-Danlos syndrome (EDS) derived fibroblast cell line. Both control and WM115 and WM266.4 samples were detached by trypsinization, washed twice with PBS, centrifuged for 15 minutes at 1200 x g (4°C), and then frozen at -70°C in a Guanidine-Iso-Thiocyanate solution (2×10^6^ cells/sample). RNA extraction was performed as described by Chomczyńsky and Sacchi [41], with slight modifications, and resuspended in distilled sterile water. RNA purity and concentration were determined both spectrophotometrically and electrophoretically.

For qualitative RT-PCR analysis, we designed an expression panel including different pro-angiogenic factors, cell-cell adhesion molecules, and matrix-metallo proteinases. Of note, we analyzed two out of the 6 possible transcripts of the melanoma adhesion molecule MCAM/MUC 18 gene: the short isoform, widely expressed by endothelial cells, and the long isoform, more melanoma-specific [11, 42–44]. Two micrograms of total RNA and 2.5 units of Moloney Murine Leukemia virus reverse transcriptase [45] (Applied BioSystems, Roche Molecular Systems, Inc., Branchburh, New Jersey, USA) were applied in all RT-PCR experiment, according to the manufacturer's instructions. For the generation of the first strand cDNA, the reaction mix contained 2.5 μM oligo d(T)_16_, 5 mM MgCl_2_, 1 μM dNTPs, 1 unit of RNase Inhibitor (Applied BioSystems) during 1 h incubation at 42°C. A 2μl aliquot of cDNA was used for single step PCR for all genes, with the exception of MCAM/MUC 18 that included subsequent nested PCR. Primer sequences and PCR conditions are reported in detail in Supplementary Information. The resulting nested products (25 μl) were analyzed on a 1.8% agarose gel. RNA integrity was checked electrophoretically, and quality of cDNA was controlled by amplification of housekeeping genes such as β2-microglobulin. The level of gene expression was evaluated by densitometric analysis through the ImageJ software, and normalized with the PCR product of the housekeeping β_2_-microglobulin gene, coamplified in the same experiment. All RT-PCR experiments were performed in triplicate. Under these conditions, gross quantitative estimations of mRNA expression could be detected.

### Generation and analysis of 3D multicellular tumor spheroids

Spheroids were generated through the liquid-overlay technique. Briefly, 100 μl/well of cell suspension at optimized densities (0.5×10^4^ cells/ml for WM115 and 0.5×10^3^ for WM266.4), were dispensed onto 96-well flat-bottomed plates (Corning B.V. Life Sciences), pre-coated with 1.5% agarose (wt/vol, Serva ELECTROPHORESIS) and incubated 4 days at 37°C, in a 5% CO_2_ humidified atmosphere. Spheroids treatment was performed by adding MC3181 and NBDHEX at a final concentration ranging from 0.005 to 50 μM (final incubation volume, 200 μl). Fifty percent of the medium in each well was replenished 48 hours after treatment and at day, 4, 6, 10 and 14. Spheroid size was measured up to 17 days by phase contrast imaging (10X magnification, 3X digital magnification) using a Nikon ECLIPSE TS100 inverted microscope (Nikon Instruments S.p.A, Florence, Italy), equipped with a digital camera. Images were analyzed by the ImageJ software. The radius of each tumor spheroid was used to calculate the volume (μm^3^):

$$\text{V}=4/3{\pi \text{ r}}^{\text{3}}$$

Spheroid growth is expressed as percentage of the volume measured in the control spheroid at the beginning of the treatment (day 0). Values are reported as means ± SD of three separate experiments, each performed in quadruplicate.

MTS assays were performed using the CellTiter Aqueous OneSolution kit (Promega Milan, Italy) according to the manufacturer's instructions. Spheroids viability is expressed as percentage of the absorbance measured in the control cells. Results are presented as means ± SD of three separate experiments, each performed in quadruplicate.

### Migration and invasion assay of 2D monolayer cultures and 3D multicellular tumor spheroids

Cell migration was performed using Boyden chambers with an 8.0 μm pore size (Corning). WM266.4, WM115 (5×10^4^ cell/well) and SK-MEL-5 cells (7.5×10^4^ cell/well) were suspended in FBS-free media and loaded into the upper chamber, in the absence or presence of increasing MC3181 concentrations. The lower chamber was filled with complete medium supplemented with 20% FBS. For the invasion assay, the transwell membranes were previously coated with 5 μg of Matrigel (BD Biosciences, Milan, Italy). After 48-hour incubation (37°C; 5% CO_2_), cells adherent to the underside of the filters were fixed and permeabilized with 70% ethanol, washed with PBS, stained with 0.25% crystal violet (Serva ELECTROPHORESIS), and counted. Four random fields at magnification 20X (3X digital camera) were counted. Percent invasion and invasion index were calculated using the following equations:

$$\%Invasion=\frac{\begin{array}{l} Meanofcellsinvadingthrough\\ Matrigelcoatedinsertmembrane \end{array}}{\begin{array}{l} Meanofcellsmigratingthrough\\ controlinsertmembrane \end{array}}\times 100$$

$$\text{Invasion Index}=\frac{\%Invasiontestcells}{\%Invasioncontrolcells}$$

Results represent the means ± SD of three independent experiments.

In the spheroid invasion assay, tumor spheroids of about 330 μm diameter were transferred in a 96-well U-bottomed plate and embedded in 0.5 mg/ml of type I collagen. After collagen solidification, 100 μl of culture medium was added to the top. When present, MC3181 (0.04-1 μM) was added to both the type I collagen and the overlaid medium. Images were captured with the inverted microscope Nikon ECLIPSE TS100 after 24 and 48 hours MC3181 incubation and analyzed through the ImageJ software. Values are expressed as means ± SD of two independent experiments, each performed in quintuplicate.

### Western blot analysis

At different time points after treatment, WM115 and WM266.4 cells were harvested, washed in PBS and suspended in lysis buffer containing 50 mM Tris–HCl (pH 7.4), 1 mM EDTA, 1 mM EGTA, 1% Triton X-100, 10 mM NaF, 1 mM Na_3_VO_4_, and protease inhibitors (Sigma–Aldrich). After 30 minutes incubation on ice, the samples were centrifuged at 13.000×g for 20 min (4°C), after which the protein concentration of the supernatant was determined using the Lowry colorimetric assay. Proteins (50 μg) were separated on 12% SDS-polyacrylamide gel and transferred onto an Immobilon-PVDF Transfer Membrane (Millipore, Billerica, MA). For immunodetection, the following primary antibodies were used: anti-phospho-JNK (Thr183/Tyr185) (Cell Signaling, Beverly, MA, USA), anti-JNK (Cell Signaling), anti-phospho-ERK1&2 (Tyr185/187; Invitrogen, Camarillo, CA), anti-ERK1&2 (Santa Cruz Biotechnology, Santa Cruz, CA), anti-phospho-p38 (Thr180/Thr182; Cell Signaling), anti-p38 (Cell Signaling), anti-AP2 (OriGene, Rockville, MD, USA), anti-CD146 (OriGene), anti-VEGF (OriGene), anti-N-cadherin (Abcam, Cambridge, UK), anti-LC3 (Novus Biologicals, Co, USA) and anti-β actin (Sigma-Aldrich) as loading control. Anti-rabbit or anti-mouse secondary antibodies (Cell Signaling) were revealed with the ECL LiteAblot Extend (EuroClone). Band intensities were measured by the ImageJ software. Data are presented as means of arbitrary units ± SD resulting from three independent experiments.

### *In vivo* murine melanoma lung metastasis model and treatment

Procedures involving animals and their care were in conformity with Institutional Guidelines (D.L. 116/92 and subsequent implementing circulars), and experimental protocols were approved by the local Ethical Committee of Padova University (CEASA). During *in vivo* experiments, animals in all experimental groups were examined daily for a decrease in physical activity and other signs of disease or drug toxicity; severely ill animals were euthanized by carbon dioxide overdose.

Six to eight week-old male C57BL/6 (B6) inbred mice (H-2^b^) were purchased from Charles River Laboratories (Calco, Italy), and housed in our Specific Pathogen Free (SPF) animal facility. B16-F10 cells were suspended in PBS and checked for viability using trypan blue staining. Only when cell viability was > 90% the cell batch was considered for injection. B16-F10 cells (1×10^5^) were injected intravenously into syngeneic C57BL/6J mice, which were then divided randomly into 2 groups (6 mice/group). Eight hours later, MC3181 was dissolved in PBS and administered orally according to a q1dx6 schedule for 2 weeks at a dose of 8 mg/kg/day. Control group received PBS. Mice were then sacrificed after two weeks of treatment, lungs were harvested, rinsed in water, and fixed in Bouin's solution. Surface metastases were then counted with the aid of a dissecting microscope.

### Statistical analysis

Statistical analyses have been performed using the Statistical Package for the Social Sciences Windows, version 15.0 (SPSS, Chicago, Illinois, USA). Descriptive statistics consisted of the mean ± SD for parameters with gaussian distributions (Kolgomorov-Smirnov test). The equality of the variances was confirmed by the Levene's Test. ANOVA one-way followed by Dunnett's test was used for multiple comparison among treatment groups (>2) and control. A P value < 0.05 was considered statistically significant.

## SUPPLEMENTARY MATERIALS FIGURES AND TABLES


