# Supplementary material for: The nitrobenzoxadiazole derivative MC3181 blocks melanoma invasion and metastasis
Source: Oncotarget. 2017 Jan 17;8(9):15520–38. doi: 10.18632/oncotarget.14690 (PMC5362503; doi:10.18632/oncotarget.14690)
Supplement: Supplementary file 1 [file oncotarget-08-15520-s001.pdf]

# The nitrobenzoxadiazole derivative MC3181 blocks melanoma invasion and metastasis

## SUPPLEMENTARY DATA

## SUPPLEMENTARY METHODS

### Reverse transcriptase-polymerase chain reaction (RT-PCR)

Primer sequences and PCR conditions were reported in international literature or designed from the predicted sequence using Gene Scan program (http://genes.mit.edu/genscan.HTML), and were synthesized by Tema Ricerca, Inc. Sequences were as follows: FGF2 (basic Fibroblast Growth Factor) sense AGCAGAAGAGAGAGG AGTTGTGTC and FGF2 antisense [1] CCCAGGTCCTGTTTTGGATCCAAG, VEGF (Vascular Endothelial Growth Factor) sense TCGGGCCTCCGAAAC CATGAACT and VEGF antisense TCCTGGTGAGAGATCTGGTTCCC [2]; MCAM/MUC18 sense GTCATCTTCCGT GTGCGCCA and MCAM/MUC18 antisense GTAGCGACCTC CTCAGGCTCCTTA; MCAM/MUC 18 sense nested GTCATCCGGTGCGCCA and MCAM/MUC18 antisense nested GTA GCG ACC TCC TCA GGC TCC TTA [3]; MCAM/MUC 18 long isoform sense CCCTCACACCAGACTCCAAC and MCAM/MUC 18 long isoform antisense GTTCGCTCTTAC; MCAM/MUC 18 long isoform nested sense ACG GGGGCA CGTTGGATTG and MCAM/MUC 18 long isoform nested antisense GTAGCGTGATCTCCTGCTTCC; MCAM/MUC 18 short isoform sense TCA TACCAGAGCCAACAGCA and MCAM/MUC 18 short isoform antisense CTCTCCAT TCCTGCTTCCC (common to first and nested PCR too); MCAM/MUC 18 short isoform sense nested AGAGAGAAAGCTGCCGGAG; CDH1 (epithelial cadherin) sense GACCAGGACTATG ACTACTTGAAACG and CDH1 antisense ATCTG CAAGGTGCTGGGTGAACCTT; CDH2 (neural cadherin) sense CTC CTA TGA GTG GAA CAG GAA CG and CDH2 antisense TTG GAT CAA TGT CAT AAT CAA GTG CTG TA [4]; CDH5 (vascular endothelial cadherin) sense CACTGGAACCCCCACAGGAAAAGA and CDH5 antisense GGACAGCGTTCTCACACACTTTGG; MMP-2 (matrix metalloproteinases) sense CCTGCCCC TCCCTTCAACCA and MMP2-antisense GTTTCCGCT TCTGGCTGGGTC [2]; MMP-9 sense CGGAGTGGCAGGGGGAAGATG and MMP-9 antisense CTCACGCGCCAGTAGAAGCG. Amplification conditions were as follows: a hot start denaturation of 15 min at 94°C was always performed, followed by 40 cycles of 1 min at 94°C, 1 min of annealing at a temperature specific for each gene, and 1 min at 72°C. Annealing temperatures for each gene expression assay were as follows: VEGF at 64°C, FGF2 and CDH2 at 60°C, MMP-2 and MMP-9 at 59°C, CDH1 and CDH5 at 58°C. MCAM/MUC18 conditions for both first round and nested PCR were as follows: 94°C for 1 min, 52°C for 1 min and 72°C for 1 min for 40 /30 cycles. A hot-start Taq polymerase was employed in each amplification.

## REFERENCES


1. Hussong JW, Rodgers GM, Shami PJ. Evidence of increased angiogenesis in patients with acute myeloid leukemia. Blood. 2000; 95:309-313.
2. Etoh T, Inoue H, Tanaka S, Barnard GF, Kitano S, Mori M. Angiopoietin-2 is related to tumor angiogenesis in gastric carcinoma: possible *in vivo* regulation via induction of proteases. Cancer research. 2001; 61:2145-2153.
3. Hoon DS, Wang Y, Dale PS, Conrad AJ, Schmid P, Garrison D, Kuo C, Foshag LJ, Nizze AJ, Morton DL. Detection of occult melanoma cells in blood with a multiple-marker polymerase chain reaction assay. Journal of clinical oncology: official journal of the American Society of Clinical Oncology. 1995; 13:2109-2116.
4. Hagen RM, Rhodes A, Oxley J, Ladomery MR. A M-MLV reverse transcriptase with reduced RNaseH activity allows greater sensitivity of gene expression detection in formalin fixed and paraffin embedded prostate cancer samples. Exp Mol Pathol. 2013; 95:98-104.

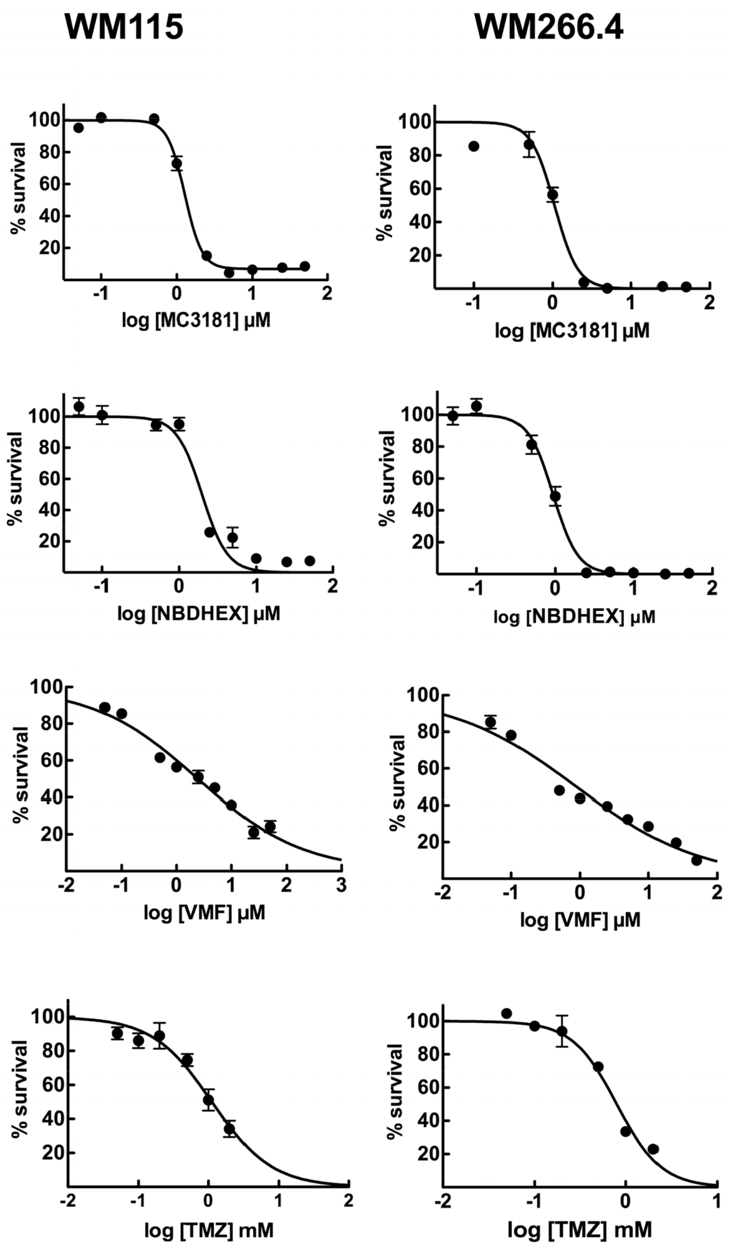


**Supplementary Figure 1: Concentration–response curves of MC3181, NBDHEX, vemurafenib (VMF) and temozolomide (TMZ).** WM115 and WM266.4 human melanoma cells were incubated with increasing concentrations of each drug, and cell growth was evaluated 48 hours later by the SRB assay. $IC_{50}$ values were obtained from the concentration–response curves from three independent experiments, and are reported in Table 1 as means ± SD.

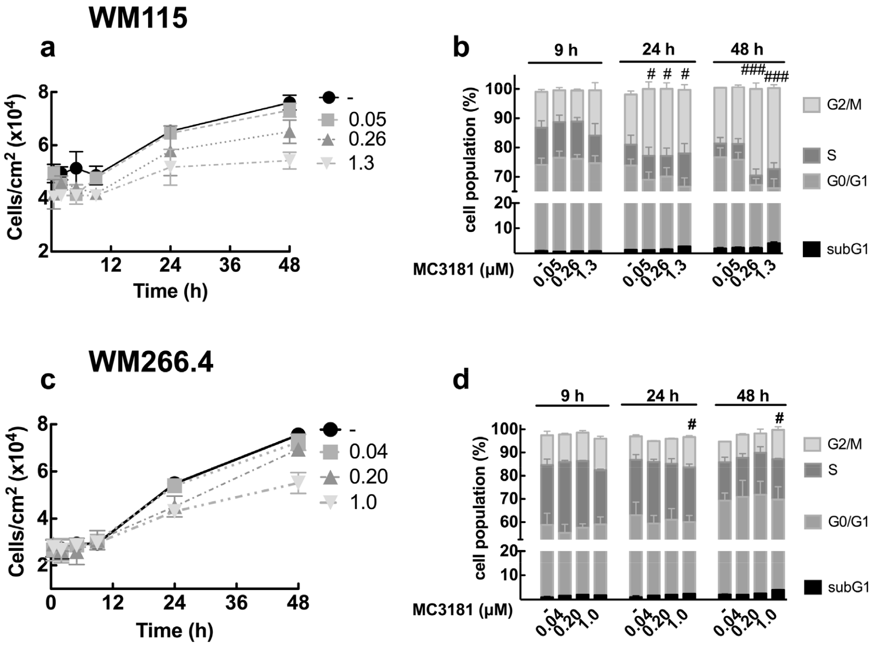


**Supplementary Figure 2: Effect of low concentrations of MC3181 on WM115 and WM266.4 cells.** Cells were treated for 48 hours with concentrations of MC3181 that matched their respective $IC_{50}$ values or 1/5 and 1/25 of their $IC_{50}$s. **a.** WM115 and **c.** WM266.4 cell growth curves. **b.** WM115 and **d.** WM266.4 cell cycle analysis performed at various time points during exposure to different concentrations of MC3181.

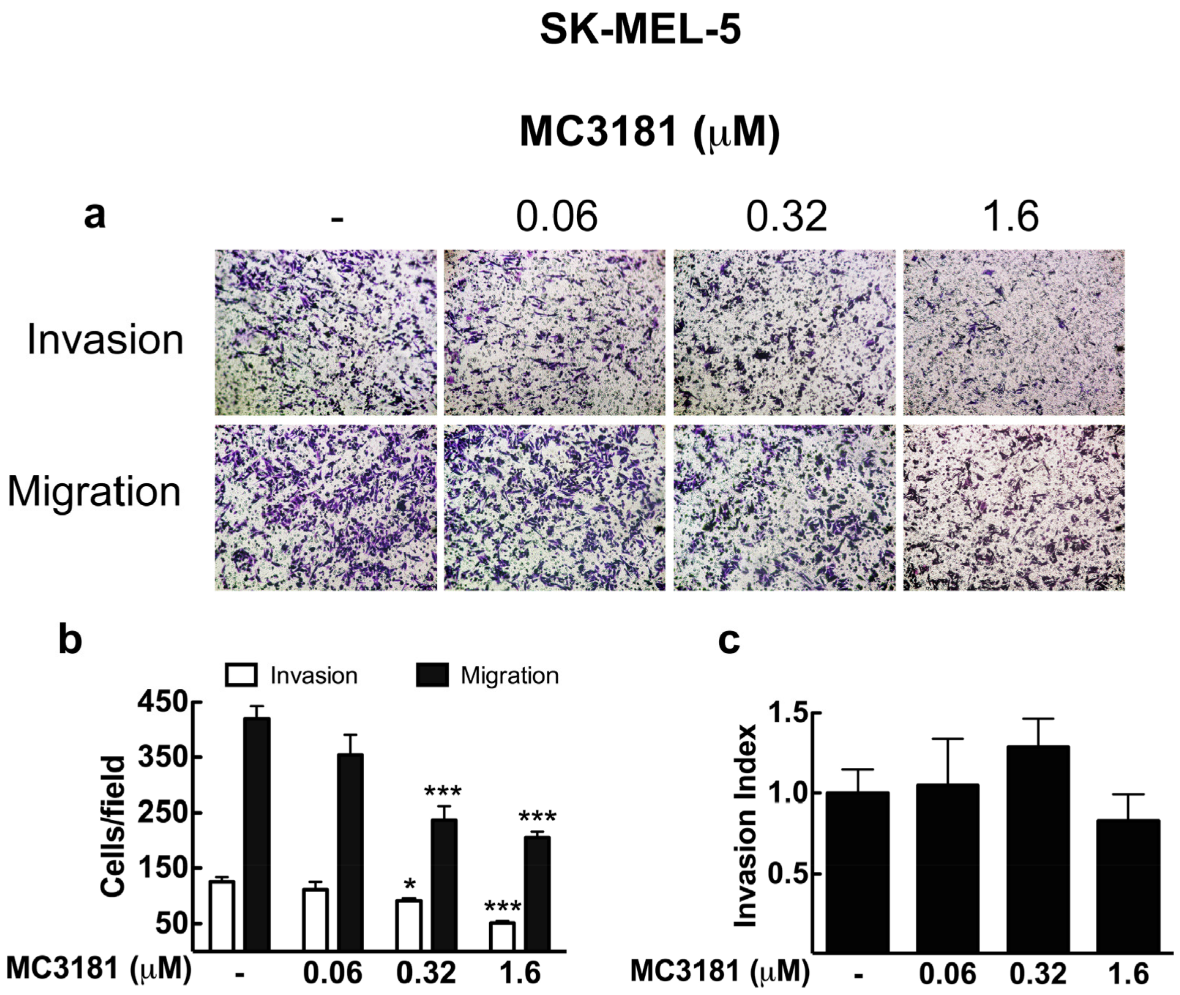


**Supplementary Figure 3: MC3181 blocks both cell invasion and migration of SK-MEL-5 melanoma cells.** The SK-MEL-5 cell line was assayed for *in vitro* invasion and migration using Boyden chambers without coating (migration) or coated with 5 μg of Matrigel. After 48 hours of treatment with graded MC3181 concentrations, the migrated and invaded cells were stained with crystal violet and counted. **a.** Representative phase contrast images (10X magnification, 3X digital magnification) of SK-MEL-5 cells. **b.** Migrated/Invaded SK-MEL-5 cells. **c.** Invasion index was calculated as the invasion percentage of treated cells divided by the invasion percentage of non-treated cells (see equations 2 and 3 in the “Materials and Methods” section).

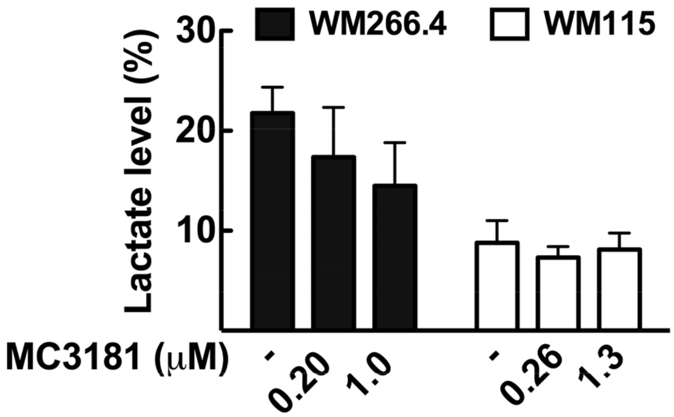


**Supplementary Figure 4: High resolution $^{1}$H-NMR analysis of WM266.4 and WM115 intracellular metabolome.** The experiment was performed on both cell lines after a 48-hours treatment with equiactive concentrations of MC3181 (0.26 and 1.3 μM for WM115, 0.20 and 1.0 μM for WM266.4). Lactate is reported as percentage of total metabolites involved in glucose, phospholipid and energetic metabolism.

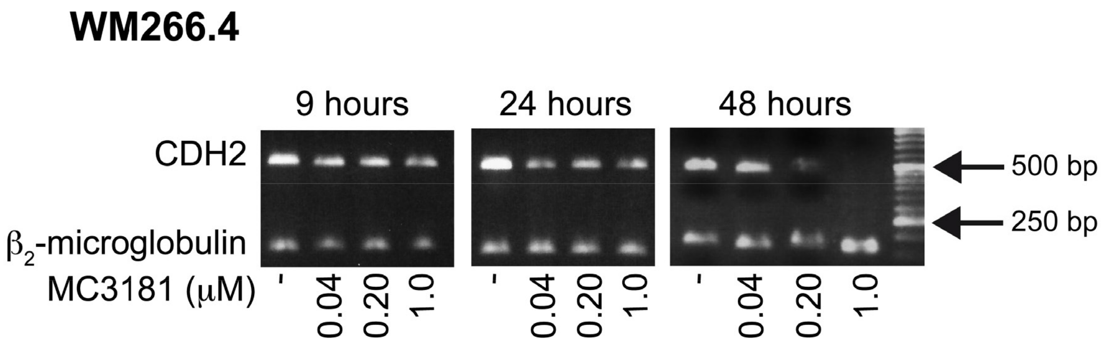


**Supplementary Figure 5: RT-PCR analysis of CDH2 mRNA in the WM266.4 cell line.** Total RNA was extracted from the cells and then reverse-transcribed by specific priming to single-stranded cDNA. The cDNA was amplified by PCR as described in the "Materials and Methods" section. PCR products were electrophoresed on a 1.8% agarose gel, stained with ethidium bromide, and the relative intensity against $\beta_2$-microglobulin was evaluated by the ImageJ software. Molecular weight markers were loaded in the last lane.

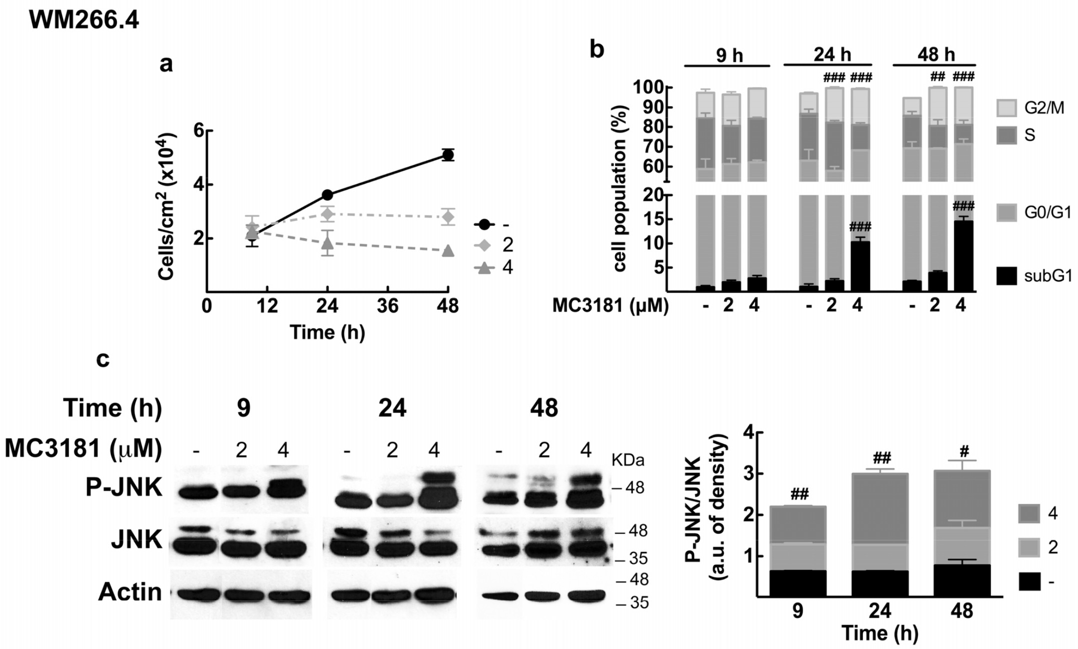


**Supplementary Figure 6: Treatment of WM266.4 cells with high concentrations of MC3181.** The compound induced cell cycle block and prolonged phospho-activation of JNK. Cells were incubated up to 48 hours with 2 and 4 μM MC3181, i.e. concentrations corresponding to 2- and 4-fold their $IC_{50}$ value. **a.** WM266.4 cell growth curves and **b.** cell cycle analysis performed at various time points during drug exposure. **c.** Immunoblot (left) and densitometric analysis (right) of P-JNK revealed a sustained increase of JNK activation after 9 hours of treatment; this increase lasted up to 48 hours. Phosphorylated and non-phosphorylated protein levels were quantitated by densitometry and normalized to their respective β-actin bands; data represent means ± SD of three independent experiments. Significant increase: $^{\#}$ $P < 0.05$, $^{\#\#}P < 0.005$, $^{\#\#\#}P < 0.0005$.

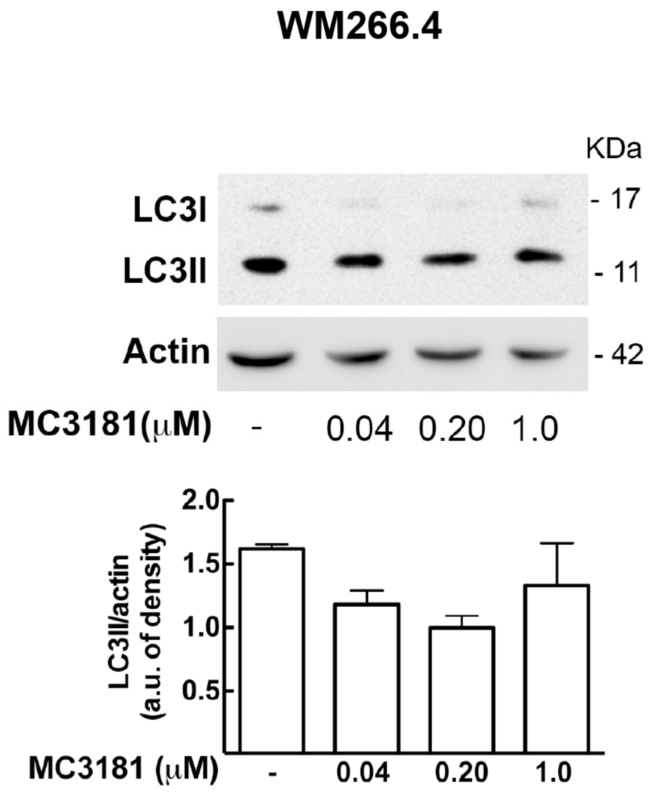


**Supplementary Figure 7: Effect of subtoxic concentrations of MC3181 on the basal autophagy of the WM266.4 cell line.** Cells were cultured for 48 hours, treated with low concentrations of MC3181 (i.e. 0.04, 0.2 and 1.0 μM) for additional 48 hours, and then analyzed by western blotting with anti-LC3 and anti-β-actin antibodies. Treatment with low concentrations of MC3181 did not significantly affect the amount of the autophagosome-associated LC3-II protein.

**Supplementary Table 1A: Relative quantification of intracellular metabolites (metabolite percentage relative to total metabolites, mean ± SEM for n ≥ 3) involved in glucose, phospholipid and energetic metabolism in WM266.4 cells following a 48-hour exposure to MC3181**

| Metabolite (%) | MC3181 (μM) | | |
|---|---|---|---|
| | - | 0.20 | 1.0 |
| **Taurine** | 3.64 ± 0.25 | 3.67 ± 0.49 | 2.73 ± 0.96 |
| **Total creatine** | 6.15 ± 0.32 | 6.95 ± 0.31 | 7.03 ± 0.11 |
| **Aspartate** | 2.72 ± 0.45 | 2.76 ± 0.42 | 3.11 ± 0.47 |
| **Total glutathione** | 4.05 ± 0.47 | 4.52 ± 0.49 | 5.17 ± 0.48 |
| **Succinate** | 0.75 ± 0.12 | 0.69 ± 0.09 | 0.73 ± 0.05 |
| **Glutamate** | 5.34 ± 0.30 | 6.04 ± 0.61 | 6.30 ± 0.52 |
| **Glx*** | 15.04 ± 1.26 | 16.36 ± 1.53 | 17.04 ± 1.01 |
| **Acetate** | 4.17 ± 0.66 | 3.81 ± 0.21 | 4.23 ± 0.61 |
| **Alanine** | 3.60 ± 0.81 | 3.24 ± 0.74 | 3.01 ± 0.58 |
| **Lactate** | 21.76 ± 2.60 | 17.36 ± 4.98 | 14.50 ± 4.32 |
| **Isoleucine** | 0.90 ± 0.16 | 0.94 ± 0.15 | 0.90 ± 0.14 |
| **Glycerophosphocholine** | 0.41 ± 0.05 | 1.75 ± 1.21 | 1.34 ± 0.66 |
| **Phosphocholine** | 4.99± 0.87 | 6.27 ± 1.16 | 7.70 ± 1.39 |
| **Choline** | 0.55 ± 0.20 | 0.40 ± 0.13 | 0.52 ± 0.35 |
| **ATP plus ADP** | 1.08 ± 0.09 | 1.01 ± 0.015 | 1.07 ± 0.03 |

*Glx: glutamine plus glutamate and glutathione

**Supplementary Table 1B: Relative quantification of intracellular metabolites (metabolite percentage relative to total metabolites, mean ± maximum deviation; n = 2) involved in glucose, phospholipid and energetic metabolism in WM115 cells following a 48-hour exposure to MC3181**

| Metabolite (%) | MC3181 (µM) | | |
|---|---|---|---|
| | - | 0.26 | 1.3 |
| **Taurine** | 2.25 ± 0.53 | 1.86 ± 0.13 | 1.72 ± 0.01 |
| **Total creatine** | 3.53 ± 0.41 | 3.39 ± 0.27 | 3.47 ± 0.09 |
| **Aspartate** | 3.70 ± 0.57 | 4.11 ± 0.28 | 3.87 ± 0.32 |
| **Total glutathione** | 5.99 ± 0.01 | 6.42 ± 0.31 | 7.16 ± 0.01 |
| **Succinate** | 0.70 ± 0.02 | 0.77 ± 0.07 | 0.76 ± 0.06 |
| **Glutamate** | 8.74 ± 0.31 | 8.31 ± 0.36 | 8.32 ± 0.25 |
| **Glx*** | 21.16 ± 0.92 | 21.39 ± 0.06 | 21.26 ± 0.42 |
| **Acetate** | 4.08 ± 2.04 | 2.85 ± 1.83 | 2.15 ± 1.57 |
| **Alanine** | 2.64 ± 0.36 | 2.91 ± 0.51 | 2.75 ± 0.37 |
| **Lactate** | 8.79 ± 2.22 | 7.31 ± 1.10 | 8.12 ± 1.66 |
| **Isoleucine** | 0.82 ± 0.07 | 0.88 ± 0.14 | 0.82 ± 0.09 |
| **Glycerophosphocholine** | 3.08 ± 1.54 | 1.94 ± 0.97 | 2.35 ± 0.21 |
| **Phosphocholine** | 8.39± 0.84 | 8.35 ± 0.53 | 9.42 ± 1.23 |
| **Choline** | 0.44 ± 0.25 | 0.99 ± 0.21 | 0.32 ± 0.22 |
| **ATP plus ADP** | 0.89 ± 0.06 | 1.23 ± 0.18 | 1.21 ± 0.18 |

*Glx: glutamine plus glutamate and glutathione
